# Bridging the Gap: Assessing Public Awareness of Psychological Factors Influencing Coronary Heart Disease Outcomes in Makkah, Saudi Arabia

**DOI:** 10.7759/cureus.51637

**Published:** 2024-01-04

**Authors:** Hadeel A AlGhamdi, Ghadi M Alhazmi, Haifa O Alsharif, Noran A Addas, Abeer Shaker Elmoursy Ali, Wesam A Nasif

**Affiliations:** 1 Medicine and Surgery, Faculty of Medicine, Umm Al-Qura University, Makkah, SAU; 2 Pathology, Faculty of Medicine, Umm Al-Qura University, Makkah, SAU; 3 Department of Biochemistry, College of Medicine, Umm Al-Qura University, Makkah, SAU; 4 Molecular Biology Department, Genetic Engineering and Biotechnology Research Institute, Sadat City University, Sadat City, Cairo, EGY

**Keywords:** quality of life, stress, psychological impact, coronary artery disease (cad), coronary heart disease (chd)

## Abstract

Background: Coronary heart disease (CHD) is a leading cause of death globally, and psychological factors are increasingly recognized as playing a significant role in its prognosis. This study aimed to assess the public's awareness of psychological factors affecting CHD prognosis in Makkah, Saudi Arabia.

Methods: A cross-sectional online survey was conducted with 385 participants recruited randomly. The survey collected data on sociodemographic characteristics and respondents' opinions regarding the effects of negative and positive psychological factors on CHD prognosis, including differences based on age and gender.

Results: The majority of participants (96.9%) agreed that negative psychological factors, such as stress (80.5%), anxiety (76.9%), and depression (67.5%), influence CHD prognosis. Positive factors like satisfaction (79.2%) and happiness (75.6%) were also recognized as influential. Participants aged 30-50 and over 50 demonstrated higher awareness of the link between psychological factors and CHD compared to those under 30. No significant gender differences were observed in knowledge levels.

Conclusion: This study suggests that the Makkah population has a good understanding of the impact of psychological factors on CHD prognosis. Integrating this knowledge into comprehensive health education programs could benefit CHD prevention, management, and prognosis in the region. Future research should explore broader populations and utilize diverse methodologies to refine and generalize these findings.

## Introduction

While cardiovascular illnesses remain the leading cause of death worldwide, claiming millions of lives annually [1], coronary heart disease (CHD) stands out as the most prevalent culprit within this category [2]. This debilitating condition, also known as atherosclerotic cardiovascular disease (ACD) or coronary artery disease (CAD), involves the progressive buildup of plaque in the arteries that supply the heart [3]. Over time, these cholesterol deposits narrow the arterial lumen, restricting blood flow and leading to the potentially fatal symptom of atherosclerosis [3]. Chest pain, weakness, nausea, and breathlessness are just a few of the warning signs that may precede heart failure due to CHD progression [3]. While established risk factors like physical inactivity, unhealthy diet, and smoking play a crucial role [4], recent research has shed light on a less-explored dimension: the impact of psychological factors on CHD progression and prognosis. The pathogenesis of CAD encompasses a complex interplay between modifiable and non-modifiable risk factors. Among the latter, increasing age, male sex, and pre-existing medical conditions such as hypertension, diabetes mellitus, hypercholesterolemia, and chronic kidney disease play a substantial role in disease initiation and progression [5]. Moreover, the clinical presentation of CAD is heterogeneous, classified into three distinct entities: stable angina, characterized by stable exertional chest pain; unstable angina, exhibiting unpredictable and potentially threatening chest discomfort; and myocardial infarction, representing the culmination of CAD in the form of acute tissue damage due to coronary occlusion [6].

The complex interplay of factors beyond lifestyle choices appears to influence the development of CAD. Notably, the quality of social relationships, coupled with mental health struggles like anxiety, stress, and inadequate sleep, can contribute to its onset [7,8]. Interestingly, individuals at risk for CAD often exhibit a combination of these factors, with over 70% having multiple identifiable risk markers. However, a small portion of the overall population, estimated at 2-7%, may develop CAD even without readily apparent risk factors [9].

The dance between psychological well-being and CHD is complex. While stress, depression, and anxiety can act as unwelcome partners, amplifying the disease and hindering treatment effectiveness, happiness, satisfaction, and social support can be powerful allies, bolstering resilience and promoting positive outcomes [10]. Understanding this intricate interplay is crucial for optimizing CHD management, where addressing both the physical and emotional aspects becomes the key to unlocking a brighter future for patients.

One explanation for this is that psychological factors can contribute to unhealthy lifestyle behaviors, such as smoking, poor diet, and lack of exercise, which are major CHD risk factors [11]. Furthermore, psychological factors can impact CHD management and treatment. For example, individuals with depression or anxiety may be less likely to adhere to medication and lifestyle recommendations, which can result in a poorer prognosis [12].

Underscoring the complex interplay between mental health and heart health, Khayyam-Nekouei's 2013 Iranian study demonstrated that individuals experiencing high levels of stress, anxiety, depression, and social isolation are not only more susceptible to developing CHD but also face a harsher prognosis following a cardiac event [10,13].

It is crucial to address psychological aspects during management and treatment to enhance outcomes and quality of life for those who have CHD [14]. The aim of the current study is to examine potential variations in public knowledge and understanding of the psychological dimension of CHD based on sociodemographic factors like age and gender.

## Materials and methods

Study population

A cross-sectional study of the population of the city of Makkah was conducted between August 2022 and June 2023 in western Saudi Arabia. Data concerning a sample of 385 individuals were collected randomly using self-administered questionnaire forms. The sample size was calculated using the sample size equation:

Sample size (n)= [DEFF x Np (1-p)]/[d^2^/Z^2^_1-a/2_x(N-1)+px(1-p)]

where n indicates the sample size, N is the study population of Makkah City (approximately 2,114,675) (General Authority for Statistics KSA, 2022), and p is the largest percentage of any community's properties that have been surveyed, which was assumed to be 50%. The hypothesized percentage of the specific outcome frequency in the population (p) may be counted as follows: 50%+/-five. Confidence limits as a percentage of 100 (absolute +/-) (d) were set at 5%. Moreover, the design 12 effect for cluster surveys (DEEF) was set at number one.

Using this formula, the required sample size was determined, resulting in a sample of 385 participants; this resulted in 95% and 5% confidence levels, with the latter representing the minimum acceptable limit. Future research requires a larger sample size to compensate for any possible data loss.

Individuals above the age of 18 years old with a history of CHD resident in Makkah city. Individuals with specific medical conditions directly affecting psychological factors or CHD prognosis (e.g., uncontrolled hypertension, substance abuse disorders) are excluded from the study.

This study was approved by the institutional review board (IRB) of MOH, Makkah, Saudi Arabia, and Umm Al-Qura University (UQU) under approval no.: HAPO-02-K-012-2022-08-1162.

Questionnaire investigating the Makkah population’s awareness of psychological factors associated with CHD prognosis

An online survey was used to determine and measure the Makkah population's knowledge regarding the effects of psychological factors on CHD prognosis. The chosen study participants included Saudi and non-Saudi males and females older than 18 years from Makkah City. WhatsApp was used to send our study's self-reporting questionnaire (developed using the Google Forms tool) to randomly chosen targeted participants by responding to the published questionnaire.

Data collection

The questionnaire collected data regarding sociodemographic factors, including age, gender, level of education, and marital status; response regarding the effects of negative and positive psychological factors affecting CHD prognosis, these factors’ different (negative or positive) effects on heart disease depending on age, the direct or indirect effect of psychological factors (negative or positive) on responses to treatment, and the relationship between stabilizing the psychological state and improving heart disease; detection of the most negative and positive psychological factors that affect heart disease.

The following is the questionnaire link: https://docs.google.com/forms/d/e/1FAIpQLSf92WPD3vrw4WwEiqihdG2BP3OeDgIuF3Pn6nMei7M6kQAsPQ/viewform?pli=1

Analytical statistics

RStudio (R version 4.2.2, RStudio Team 2015, RStudio: Integrated Development for R. RStudio, Inc., Boston, MA) was utilized to perform the statistical analysis. Categorical data were expressed as frequencies and percentages. Statistical differences based on participants' genders and age groups were tested using the Pearson Chi-squared test and the Fisher exact test. At p< 0.05, statistical significance was determined.

## Results

Participants’ sociodemographic characteristics

Of the 389 responses collected, we excluded four respondents who declined to participate. Therefore, data from 385 respondents were analyzed. Almost two-thirds of participants were female (69.1%) and married (60.8%). Additionally, 38.7% of respondents were aged 30 to <50 years and less than half were employed (42.9%) (Table 1).

**Table 1:** Participants’ sociodemographic characteristics

Parameter	Category	N (%)
Gender	Male	119 (30.9%)
	Female	266 (69.1%)
Age	<30	93 (24.2%)
	30 to <50	149 (38.7%)
	50 or more	143 (37.1%)
Marital status	Single	108 (28.1%)
	Married	234 (60.8%)
	Divorced	25 (6.5%)
	Widow	18 (4.7%)
Employment status	Student	88 (22.9%)
	Employed	165 (42.9%)
	Unemployed	61 (15.8%)
	Retired	71 (18.4%)

Participants' responses regarding their awareness of psychological factors’ effects on heart disease

**Figure 1:**
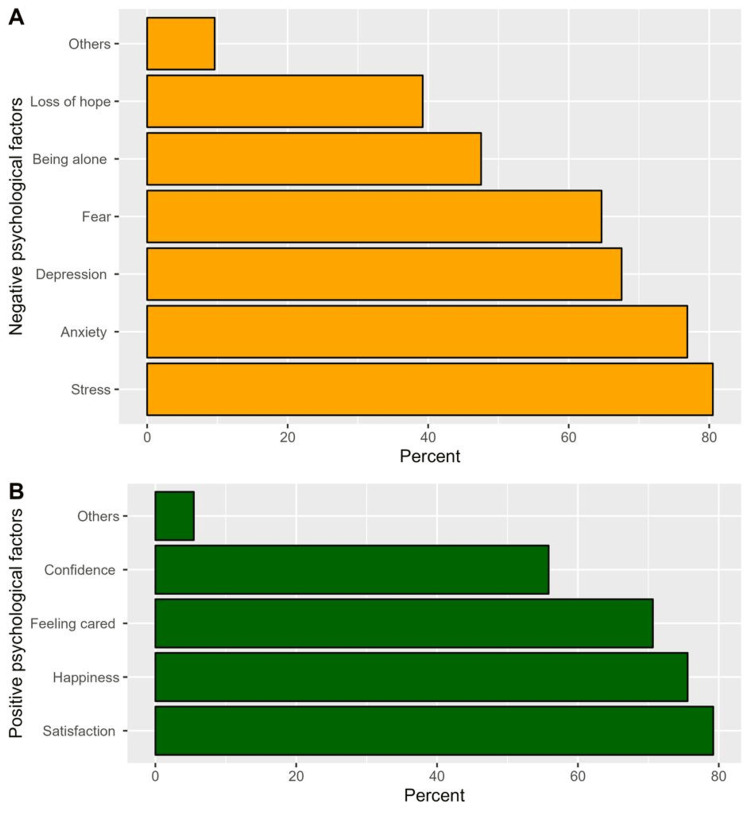
Participants’ responses regarding the most common negative and positive psychological factors that would impact heart disease prognosis

The majority of respondents agreed that negative and positive psychological factors would impact heart disease prognosis (96.9% and 87.5%, respectively, Table 2). The most common negative psychological factors that would impact heart disease prognosis that respondents identified were stress (80.5%), anxiety (76.9%), and depression (67.5%, Figure 1A). Conversely, the most frequently perceived positive psychological effects were satisfaction (79.2%), happiness (75.6%), and feeling cared for (76.6%, Figure 1B). Additionally, a significant proportion of respondents indicated that the effects of psychological factors on heart disease differ according to patients’ ages (78.3%), that psychological factors have a direct or indirect effect on responses to treatment (91.2%), and that treating and stabilizing the psychological state is one way to improve heart disease (91.2%, Table 2).

**Table 2:** Participants’ responses regarding their awareness of psychological factors’ effects on heart disease *the variable had three missing records

Parameter	No	Yes	Do not know	Total
Negative psychological factors impact heart disease prognosis	4(n) (1.0%)	373(n) (96.9%)	8(n) (2.1%)	385(n) (100%)
Positive psychological factors influence heart disease prognosis	26(n) (6.8%)	337(n) (87.5%)	22(n) (5.7%)	385(n) (100%)
Psychological factors’ effects on heart disease differ according to patients’ ages*	35(n) (9.2%)	299(n) (78.3%)	48(n) (12.6%)	382(n) (99.2%)
Psychological factors have a direct or indirect effect on the response to treatment	7(n) (1.8%)	351(n) (91.2%)	27(n) (7.0%)	385(n) (100%)
Treating and stabilizing the psychological state is one way to improve heart disease	10(n) (2.6%)	351(n) (91.2%)	24(n) (6.2%)	385(n) (100%)

Gender differences (according to participants’ responses regarding the most negative and positive psychological factors that would affect heart disease)

An analysis of gender-based differences in participants’ responses revealed that significantly higher proportions of males perceived depression to be the most negative psychological factor that influences heart disease (76.5% vs. 63.5% among females, p = 0.012), and that confidence is the most positive psychological factor that affects heart disease (67.2% vs. 50.8% among females, p = 0.003). However, no significant differences were noted in participants’ responses regarding other variables (Table 3).

**Table 3:** Gender-based differences in participants’ responses to questions related to psychological factors’ effects on heart disease

Parameter	Category	Male, N = 119	Female, N = 266	p-value
Negative psychological factors impact heart disease prognosis	No	2 (1.7%)	2 (0.8%)	0.772
	Yes	115 (96.6%)	258 (97.0%)	
	Do not know	2 (1.7%)	6 (2.3%)	
Positive psychological factors influence heart disease prognosis	No	6 (5.0%)	20 (7.5%)	0.608
	Yes	107 (89.9%)	230 (86.5%)	
	Do not know	6 (5.0%)	16 (6.0%)	
Psychological factors’ (positive or negative) effects on heart disease differ according to patients’ ages	No	13 (11.1%)	22 (8.3%)	0.587
	Yes	88 (75.2%)	211 (79.6%)	
	Do not know	16 (13.7%)	32 (12.1%)	
Psychological factors (positive or negative) have a direct or indirect effect on the response to treatment	No	4 (3.4%)	3 (1.1%)	0.114
	Yes	110 (92.4%)	241 (90.6%)	
	Do not know	5 (4.2%)	22 (8.3%)	
Treating and stabilizing the psychological state is one way to improve heart disease	No	5 (4.2%)	5 (1.9%)	0.274
	Yes	105 (88.2%)	246 (92.5%)	
	Do not know	9 (7.6%)	15 (5.6%)	
The most negative psychological factors you think will affect heart disease	Being alone	57 (47.9%)	126 (47.4%)	0.923
	Anxiety	95 (79.8%)	201 (75.6%)	0.359
	Fear	76 (63.9%)	173 (65.0%)	0.824
	Depression	91 (76.5%)	169 (63.5%)	0.012
	Stress	97 (81.5%)	213 (80.1%)	0.742
	Loss of hope	50 (42.0%)	101 (38.0%)	0.452
	Others	8 (6.7%)	29 (10.9%)	0.199
The most positive psychological factors you think will affect heart disease	Satisfaction	98 (82.4%)	207 (77.8%)	0.311
	Happiness	95 (79.8%)	196 (73.7%)	0.194
	Confidence	80 (67.2%)	135 (50.8%)	0.003
	Feeling cared for	79 (66.4%)	193 (72.6%)	0.219
	Others	7 (5.9%)	14 (5.3%)	0.805

Age differences (according to participants’ responses regarding the most common psychological factors that would affect heart disease)

Regarding age-based differences, a significantly lower proportion of participants aged <30 years (83.9%) indicated that treating and stabilizing the psychological state is one way to improve heart disease compared to those aged 30 to <50 years (93.3%) and 50 years or more (93.7%). Nevertheless, higher proportions of respondents aged <30 years indicated that depression, stress, and satisfaction were the most common psychological factors that would affect heart disease (78.5%, 84.9%, and 81.7%, respectively) compared to respondents aged 30 to <50 years (70.5%, 78.5%, and 76.5%, respectively) and 50 years or more (57.3%, 79.7%, and 80.4%, respectively); these differences were statistically significant (p = 0.037, 0.002, and 0.043, respectively, Table 4).

**Table 4:** Age-based differences in participants’ responses to questions related to psychological factors’ effects on heart disease

Parameter	Category	Age (years)			p-value
		<30, N = 93	30 to <50, N = 149	50 or more, N = 143	
Negative psychological factors impact heart disease prognosis	No	2 (2.2%)	1 (0.7%)	1 (0.7%)	0.732
	Yes	89 (95.7%)	144 (96.6%)	140 (97.9%)	
	Do not know	2 (2.2%)	4 (2.7%)	2 (1.4%)	
Positive psychological factors influence heart disease prognosis	No	6 (6.5%)	8 (5.4%)	12 (8.4%)	0.543
	Yes	79 (84.9%)	133 (89.3%)	125 (87.4%)	
	Do not know	8 (8.6%)	8 (5.4%)	6 (4.2%)	
Psychological factors’ (positive or negative) effects on heart disease differ according to patients’ ages	No	11 (12.1%)	14 (9.4%)	10 (7.0%)	0.168
	Yes	63 (69.2%)	120 (80.5%)	116 (81.7%)	
	Do not know	17 (18.7%)	15 (10.1%)	16 (11.3%)	
Psychological factors (positive or negative) have a direct or indirect effect on the response to treatment	No	4 (4.3%)	1 (0.7%)	2 (1.4%)	0.218
	Yes	85 (91.4%)	138 (92.6%)	128 (89.5%)	
	Do not know	4 (4.3%)	10 (6.7%)	13 (9.1%)	
Treating and stabilizing the psychological state is one way to improve heart disease	No	6 (6.5%)	3 (2.0%)	1 (0.7%)	0.043
	Yes	78 (83.9%)	139 (93.3%)	134 (93.7%)	
	Do not know	9 (9.7%)	7 (4.7%)	8 (5.6%)	
The most negative psychological factors you think will affect heart disease	Being alone	45 (48.4%)	66 (44.3%)	72 (50.3%)	
	Anxiety	71 (76.3%)	111 (74.5%)	114 (79.7%)	0.575
	Fear	63 (67.7%)	105 (70.5%)	81 (56.6%)	0.565
	Depression	73 (78.5%)	105 (70.5%)	82 (57.3%)	0.037
	Stress	79 (84.9%)	117 (78.5%)	114 (79.7%)	0.002
	Loss of hope	33 (35.5%)	60 (40.3%)	58 (40.6%)	0.450
	Others	4 (4.3%)	13 (8.7%)	20 (14.0%)	0.697
The most positive psychological factors you think will affect heart disease	Satisfaction	76 (81.7%)	114 (76.5%)	115 (80.4%)	0.043
	Happiness	74 (79.6%)	116 (77.9%)	101 (70.6%)	0.565
	Confidence	56 (60.2%)	84 (56.4%)	75 (52.4%)	0.210
	Feeling cared for	57 (61.3%)	106 (71.1%)	109 (76.2%)	0.495
	Others	2 (2.2%)	7 (4.7%)	12 (8.4%)	0.048

## Discussion

Several studies have established the relationship between psychological factors and CHD. Chronic work stress, depression, and anxiety are major psychological stressors linked to CHD development and progression. Work stress, characterized by physical, emotional, and mental strain from work demands, has been consistently associated with CHD in numerous studies. Similarly, depression, a prevalent mental health disorder marked by persistent sadness, hopelessness, and functional impairment, is a significant risk factor for CHD. Furthermore, anxiety, characterized by excessive worry and physical symptoms like elevated heart rate and blood pressure, has been shown to contribute to CHD development [14,15]. These psychological stressors exert their influence through various biological pathways, including inflammatory responses, hormonal imbalances, and autonomic nervous system dysregulation, ultimately increasing the risk of CHD events. The aim of the current research was to assess public awareness of psychological factors and their long-term effects on CHD prognosis in the Makkah region.

An earlier study found that failing to acknowledge psychosocial risk factors could result in insufficient preventive and treatment measures, which further raise the CHD burden [15]. Regarding this, our findings revealed that the Makkah population had a good awareness of CHD psychological risk factors; the majority of respondents were in agreement regarding negative and positive psychological factors’ influences on heart disease prognosis (96.9% and 87.5%, respectively).

Stress (80.5%), anxiety (76.9%), and depression (67.5%) were the most commonly chosen negative psychological factors by both males and females. According to the 2012 European Guidelines for Cardiovascular Disease (CVD) Prevention, these three psychological factors are recognized as risk factors for incident CVD and a worsened prognosis in known CVD patients [16].

Stress was the most commonly chosen factor; as stress is related to many aspects of daily life (work, family life, etc.), it could be the psychological factor experienced most often by the general population. Stress has several CVD risks. According to previous research, evidence suggests that elevated hair cortisol levels (a reliable chronic stress biomarker) are linked to both an increased CVD risk and less favorable prognoses for recovery and treatment. Furthermore, elevated hair cortisol levels have been associated with established cardiometabolic CVD risk factors, such as high blood pressure, diabetes, and obesity [17].

Conversely, the most commonly chosen positive psychological factors that affect CHD were satisfaction, happiness, and feeling cared for. According to a previous study, there is growing evidence that positive psychological well-being can help reduce the risk of CVD and improve longevity [18]. We believe that this similarity in the two studies’ results is due to the importance of satisfaction in all patients’ lives; therefore, satisfaction was perceived to have a major effect on disease prognosis. This result increases our awareness of positive psychological factors, and that they should be considered an important aspect of treatment plans, as well as prognosis and follow-up. It will also help families (who care for such patients) provide emotional support and contribute to the best treatment plan.

In regard to the stabilization of psychological factors and their effects on CHD, research at Cochrane Library helped evaluate psychological treatments’ impacts on CHD patients’ total mortality and cardiac morbidity in comparison to standard care, as well as participant-reported psychological outcomes for depression, anxiety, and stress. The results showed insufficient proof that psychological therapy reduces overall mortality, but it does lower the cardiac death rate and ease psychological symptoms, including anxiety, stress, and depression [19].

Furthermore, another study’s findings indicate that cardiovascular outcomes for patients with depression and anxiety are significantly improved by mental health therapies for treating anxiety and depression [20]. Considering age-based differences, our research shows that participants aged 30-50 (93.3%) and >50 (93.7%) were more knowledgeable than participants aged <30 (83.9%). Regarding gender-based differences, there was no significant difference between male and female knowledge regarding this topic (92.4% and 90.6%, respectively).

The generalizability of this study's findings is limited by its focus on the Makkah population, whose unique cultural and religious context may not reflect the views of other communities. Furthermore, potential differences in participant responses between in-person interviews and online questionnaires could introduce bias and affect the accuracy of the results. Future research addressing these limitations through diverse sampling strategies and mixed-methods approaches (combining interviews and questionnaires) would enhance the generalizability, accuracy, and precision of findings on similar topics.

## Conclusions

This study’s results show that the majority of respondents agreed that negative and positive psychological factors impact heart disease prognosis (96.9% and 87.5%, respectively). To improve CHD care throughout the Middle East, a wider assessment of the general public's awareness of psychological factors’ impacts on CHD prognosis is required. To reduce CHD’s mortality and morbidity and improve its prognosis in the future, there is a critical need to provide comprehensive health education to the general population regarding psychological factors’ impacts on CHD.

We are highly recommended to develop culturally appropriate educational campaigns highlighting the significant influence of both negative and positive psychological factors on heart disease prognosis. Training healthcare professionals in identifying and managing psychological factors impacting CHD patients, in addition to developing evidence-based interventions for addressing negative psychological factors and promoting positive ones in CHD management. Finally, conduct further research to explore the specific psychological mechanisms influencing CHD prognosis in Saudi Arabia as part of Saudi Vision 2030.
