# Correction: Bridging the Gap: Assessing Public Awareness of Psychological Factors Influencing Coronary Heart Disease Outcomes in Makkah, Saudi Arabia

**DOI:** 10.7759/cureus.c163

**Published:** 2024-03-01

**Authors:** Hadeel A AlGhamdi, Ghadi M Alhazmi, Haifa O Alsharif, Noran A Addas, Abeer Shaker Elmoursy Ali, Wesam A Nasif

**Affiliations:** 1 Medicine and Surgery, Faculty of Medicine, Umm Al-Qura University, Makkah, SAU; 2 Pathology, Faculty of Medicine, Umm Al-Qura University, Makkah, SAU; 3 Department of Biochemistry, College of Medicine, Umm Al-Qura University, Makkah, SAU; 4 Molecular Biology Department, Genetic Engineering and Biotechnology Research Institute, Sadat City University, Sadat City, Cairo, EGY

This article has been corrected at the request of the authors to change the affiliations as follows:

Hadeel A. AlGhamdi Original: Cardiology, Faculty of Medicine, Umm Al-Qura University, Makkah, SAU Corrected: Medicine and Surgery, Faculty of Medicine, Umm Al-Qura University, Makkah, SAU

Ghadi M. Alhazmi Original: Medicine and Surgery, Faculty of Medicine, Umm Al-Qura University, Makkah, SAU Corrected: Medicine and Surgery, Faculty of Medicine, Umm Al-Qura University, Makkah, SAU

Haifa O. Alsharif Original: College of Medicine, Faculty of Medicine, Umm Al-Qura University, Makkah, SAU Corrected: Medicine and Surgery, Faculty of Medicine, Umm Al-Qura University, Makkah, SAU

Noran A. Addas Original: College of Medicine, Faculty of Medicine, Umm Al-Qura University, Makkah, SAU Corrected: Medicine and Surgery, Faculty of Medicine, Umm Al-Qura University, Makkah, SAU

Abeer Shaker Elmoursy Ali Original: Pathology, Faculty of Medicine, Umm Al-Qura University, Makkah, SAU Corrected: Pathology, Faculty of Medicine, Umm Al-Qura University, Makkah, SAU

Wesam A. Nasif Original: Biochemistry, Genetic Engineering and Biotechnology Research Institute, University of Sadat City, Sadat City, EGY Corrected: Department of Biochemistry, College of Medicine, Umm Al-Qura University, Makkah, SAU; Molecular Biology Department, Genetic Engineering and Biotechnology Research Institute, Sadat City University, Sadat City, Cairo, EGY

The authors deeply regret that these errors were not identified and addressed prior to publication.

 

